# Multi-level Supportive Policy Dataset for China’s Resource-Based Cities (2003 to 2023)

**DOI:** 10.1038/s41597-025-05965-y

**Published:** 2025-10-31

**Authors:** Yanchao Feng, Tong Yan, Shilei Hu, Zhenhua Zhang

**Affiliations:** 1 Business School, Zhengzhou University, Zhengzhou, 450001 China; 2 School of Economics and Management, Harbin Institute of Technology Weihai, Weihai, 264209 China; 3 School of Economics, Lanzhou University, Lanzhou, 730000 China

**Keywords:** Environmental economics, Environmental impact

## Abstract

In China’s hierarchical governance system, the central government formulates overarching strategies and targets, while provincial, municipal, and county authorities implement them after making appropriate adjustments based on local resource endowments, administrative capabilities, and development priorities. However, existing research often uses proxy variables for policy factors, and current policy intensity indices predominantly focus on policy objectives and instruments, lacking direct, comprehensive quantification methods. To bridge this gap, this study expands the policy intensity concept to incorporate policy levels, objectives, and instruments, quantified through word frequency analysis. Using 3788 government work reports (2003–2023) across national, provincial, and prefectural levels, we construct a multidimensional supportive policy intensity index. The dataset, available in.dta and.xlsx formats, includes individual policy report inventory and intensity, and aggregated policy intensity across four administrative levels, four objectives, and five policy instruments. When integrated with micro- and macro-level data on green transformation performance and environmental pollution, this dataset offers substantial potential for interdisciplinary research.

## Background & Summary

Amid growing global challenges of climate change, resource depletion, and environmental degradation, sustainable development has emerged as a universal priority^1–5^. During China’s early economic growth, resource-based cities (RBCs) played a pivotal role in national industrialization^6,7^. Leveraging their abundant natural resources, these cities made substantial contributions to building China’s self-sufficient comprehensive industrial system and rapid national economy growth^8–11^.

However, resource depletion and worsening ecological degradation have heightened the environmental protection-resource exploitation tension^12–15^, rendering sustainable transition both urgent and inevitable^16–21^. China’s “dual carbon” targets and the broader transition toward a green, low-carbon economy have further amplified these pressures^11,22^. In response, China has developed a multi-level policy system in which the national government formulates broad strategic plans, while provincial and prefectural governments issued targeted measures tailored to local conditions. Since the early 21st century, this system has generated a series of coordinated, though occasionally overlapping, policies. These include pilot programs for transforming resource-depleted cities, support for industrial diversification, shantytown renovation, and reemployment initiatives for laid-off workers.

Table 1 provides an overview of key national-level policy documents that have guided the transformation of RBCs from 2003 to 2023. The table illustrates the evolution of policy priorities, beginning with regional revitalization strategies, progressing to system-wide planning, and most recently focusing on transformation demonstrations aligned with carbon neutrality goals. The 2013 *National Resource-Based City Sustainable Development Plan (2013–2020)* marked a watershed moment, officially designating 262 RBCs and providing a systematic framework for implementation^11,23–25^.

**Table 1:** Major national-level supportive policies for RBCs in China (2003–2023).

Time	Name of documents
Oct. 2003	Opinions on the Strategy of Revitalizing Northeast China and Other Old Industrial Bases
Sept. 2004	Planning Outline for Prospecting Replacement Resource of National Crisis Mines (2004–2010)
Aug. 2005	Integrated Solutions of Population and Development Issues in Resource-Exhausted Cities with Scientific Outlook on Development
Aug. 2007	Plan for Revitalizing Northeast China
Dec. 2007	Opinions on Promoting Sustainable Development of Resource-Based Cities
Sept. 2009	Opinions on Further Implementing the Strategy of Revitalizing Northeast China and Other Industrial Bases
Oct. 2009	Guiding Opinions on Formulating Transition Plans for Resource-Exhausted Cities
Jul. 2010	Opinions on Further Supporting Overall Revitalization of Northeast China and Other Old Industrial Bases by China Development Bank
Sept. 2010	Progress Report on Revitalizing Northeast China and Other Old Industrial Bases in 2009 and Key Tasks for the Next Phase
Nov. 2013	National Resource-Based City Sustainable Development Plan (2013–2020)
Apr. 2016	Several Opinions on Comprehensive Revitalization of Northeast China and Other Old Industrial Bases
Aug. 2016	Three-Year Rolling Implementation Plan for Revitalizing Northeast China and Other Old Industrial Bases (2016–2018)
Sept. 2016	Implementation Opinions on Supporting Industrial Transformation and Upgrading of Old Industrial Cities and Resource-Based Cities
Jan. 2017	Guiding Opinions on Strengthening Categorized Guidance and Cultivating New Momentum for Transformation and Development of Resource-Based Cities
Apr. 2017	Notice on Supporting the Construction of First-Batch Demonstration Zone for the Transformation and Upgrading of Old Industrial Cities and Resource-Based Cities
Apr. 2018	Notice on Allocating 2018 Central Government Transfer Payments to Resource-Exhausted Cities
Nov. 2021	Implementation Plan for High-Quality Development of Industrial Transformation and Upgrading Demonstration Zones in Old Industrial Cities and Resource-Based Cities During the 14th Five-Year Plan Period

While this hierarchical policy architecture seeks to balance top-down guidance with bottom-up adaptability, its coordination effectiveness remains uncertain. National policies typically set ambitious yet uniform objectives, whereas sub-national policies exhibit variations in intensity, scope, and implementation capacity. Such disparities may result in policy misalignment, inconsistent execution, and divergent regional transformation outcomes.

Policy intensity refers to the degree of policy stringency and importance, typically assessed through their objectives and instruments^26–30^. Policy objectives specify the specific issues policies aim to address, whereas policy instruments reflect the guiding norms and implementation preferences^26,30^. Within this “policy objective-policy instrument” framework, scholars have quantified policy intensity from multiple perspectives. For instance, some studies have coded policy objectives related to emission reduction and renewable energy promotion^31^, while others classify policy instruments into environmental, supply-side, and demand-side categories^32,33^. More comprehensive approaches incorporate additional dimensions such as budget, implementation, scope, and monitoring to construct composite indices^31,34,35^.

Among methodological tools for policy evaluation, the Policy Modeling Consistency Index (PMC index) has emerged as a notable framework for quantitative policy content analysis. Originally developed to assess consistency across policy components, the PMC index has been widely applied in diverse domains including land protection^36^, industry policy^37^, green development^38,39^, and public health^40,41^. However, existing PMC-based studies predominantly focused on textual consistency and qualitative scoring at national or sectoral levels, frequently overlooking the continuous measurement of policy intensity and the interplay of policy levels, objectives, and instruments. Moreover, current PMC applications seldom integrate multi-tiered datasets spanning extended time periods, consequently limiting their capacity to capture temporal dynamics and sub-national heterogeneity.

Despite increasing scholarly attention to policy evaluation, most existing policy intensity indices, whether based on PMC or alternative methodologies, remain confined to national-level analyses, overlooking substantial variations in sub-national policy implementation. For instance, the OECD’s Environmental Policy Stringency Index (EPSI) evaluates 13 policy instruments across 40 countries^42,43^, while other indices target national policy portfolios for specific countries and periods (e.g., Austria, Germany, and the UK from 1998 to 2010)^31^. These efforts typically neglect intra-national regional disparities. In reality, administrative hierarchy substantially shapes policy intensity: national policies tend to be broad and strategic^34^, whereas sub-national policies (i.e., policies at the provincial or prefectural levels) are typically more tailored to local contexts, implementing more targeted and intensive measures.

To address these gaps, this study constructs a multidimensional, multi-level supportive policy intensity index for China’s RBCs covering 2003–2023, utilizing 3788 government work reports across national, provincial, and prefectural levels. Unlike the PMC index, which typically evaluates policy consistency within a single administrative scope, our dataset integrates manual annotation with quantitative scoring across three dimensions, enabling systematic comparison of both development phases and policy tiers. Focusing on RBCs (cities with distinct economic trajectories and policy needs), we not only extend the application scenarios of policy intensity measurement but also provide a richer empirical basis for examining the role of supportive policies in resource-dependent regional transitions.

The contributions of this study are threefold. First, we develop the first quantitative dataset measuring RBCs supportive policy intensity across the complete “national-provincial-prefecture” hierarchy. Second, the dataset disaggregates policy intensity into four administrative levels, four policy objectives, and five policy instruments, providing comprehensive metrics for empirical research. Third, by integrating frequency-based text analysis with multi-level decomposition, this framework enables researchers to evaluate coordinated effects and potential misalignments within multi-level policy systems, which are largely neglected in existing literature.

## Methods

**Fig. 1:**
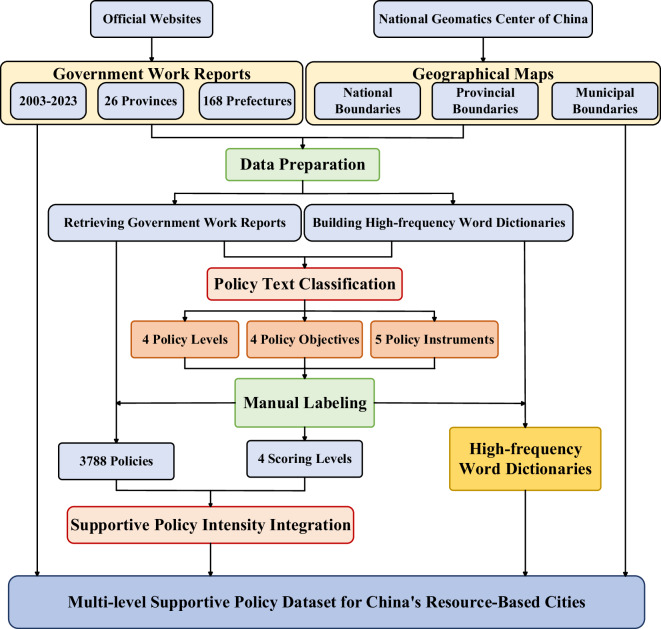
Research framework for constructing the supportive policy intensity index.

The research framework of this study comprises five parts, as illustrated in Fig. 1. We first collected and processed 3788 government work reports issued between 2003 and 2023. Based on these primary policy texts, we extended the conventional “policy objective-policy instrument” analytical model by introducing policy level as an additional dimension, thereby developing a multidimensional index of supportive policy intensity. Text mining techniques were employed to extract relevant policy expressions and compute final policy intensity scores through the integration of policy levels, objectives, and instruments.

### Data preparation

The data preparation phase establishes the basis for constructing the supportive policy intensity index. This process involves two main steps: (1) retrieval of government work reports, and (2) development of a high-frequency word dictionary.

#### Step 1: Retrieval of government work reports

This study employed Python to systematically collect government work reports from official websites of China’s central, provincial, and prefectural governments. These websites are recognized as the most authoritative and comprehensive sources for policy documentation. The sample covers 2003–2023, with 2003 selected as the starting point because it marked the beginning of enhanced policy support for RBCs. These cities underwent substantial economic reconstructing in the early 21st century while confronting growing environmental challenges. Government work reports during this period provide continuous, systematic documentation of policy actions and priorities.

We compiled a dataset of 3788 government work reports spanning multiple administrative levels: the State Council (central government), 26 provincial governments, and 168 resource-based prefectural cities.

#### Step 2: Construction of high-frequency word dictionaries

We performed text segmentation and frequency analysis on the collected reports to construct high-frequency word dictionaries for each administrative level. Using Python, Chinese word segmentation was conducted and term frequencies were extracted. To ensure the extracted terms reflect substantive policy content rather than grammatical or functional elements^35^, stop words were removed by using the publicly available “cn_stopwords” list.

Following stop-word removal, we calculated the frequency of each remaining term for the central, provincial, and prefectural-level corpora separately, generating three administrative-level-specific high-frequency word dictionaries. Each dictionary contains the most frequently used policy terms and corresponding frequencies, documenting distinctive policy discourse patterns across government tiers.

These dictionaries served as the basis for identifying keywords associated with policy objectives and policy instruments, which were then used in the subsequent quantification of policy intensity.

### Policy text classification

Following the extended “policy objective-policy instrument” analytical framework, this study categorizes supportive policy texts through three critical dimensions: policy level, objective, and instrument. These dimensions are essential for constructing a comprehensive and multidimensional measure of supportive policy intensity.

Building on the high-frequency word dictionaries generated during data preparation stage, we categorize each government work report according to its administrative level of origin. For the policy objective dimension, we identify four overarching goals commonly emphasized in RBCs supportive policies. Meanwhile, policy instruments are classified into five distinct categories. Overall, through comprehensive review of existing literature on RBCs supportive polices, we systematically developed sub-dimensions for different policy instruments and objectives. Table 2 presents the complete classification scheme.

**Table 2:** Selection of sub-dimensions across different dimensions for policy text classification.

Policy dimension	Subdimension
Policy levels	National level
	Provincial level
	Prefectural level
	County and district level
Policy objectives	Resource security
	Economic vitality
	Ecological quality
	Public wellbeing
Policy instruments	Transfer payments
	Resource compensation
	Industry support
	Price regulation
	Successor industry promotion

### Quantifying supportive policy intensity

As mentioned earlier, following the classification of policy texts into predefined categories, we incorporate policy level as an additional dimension within the extended “policy objective-policy instrument” framework to build a comprehensive index of supportive policy intensity. The index is calculated for each region and year using Eq. (1):

1 $$P{I}_{r,t}={L}_{r,t,l}\times {O}_{r,t,o}\times {I}_{r,t,i}$$

where *PI*_*r,t*_ represents the supportive policy intensity for one policy in region *r* and year *t*. *L*_*r,t,l*_ represents the intensity of policy level *l* for one policy in region *r* and year *t*. *O*_*r,t,o*_ denotes the intensity of policy objective *o* for one policy in region *r* and year *t*. *I*_*r,t,i*_ refers to the intensity of policy instrument *i* for one policy in region *r* and year *t*.

**Fig. 2:**
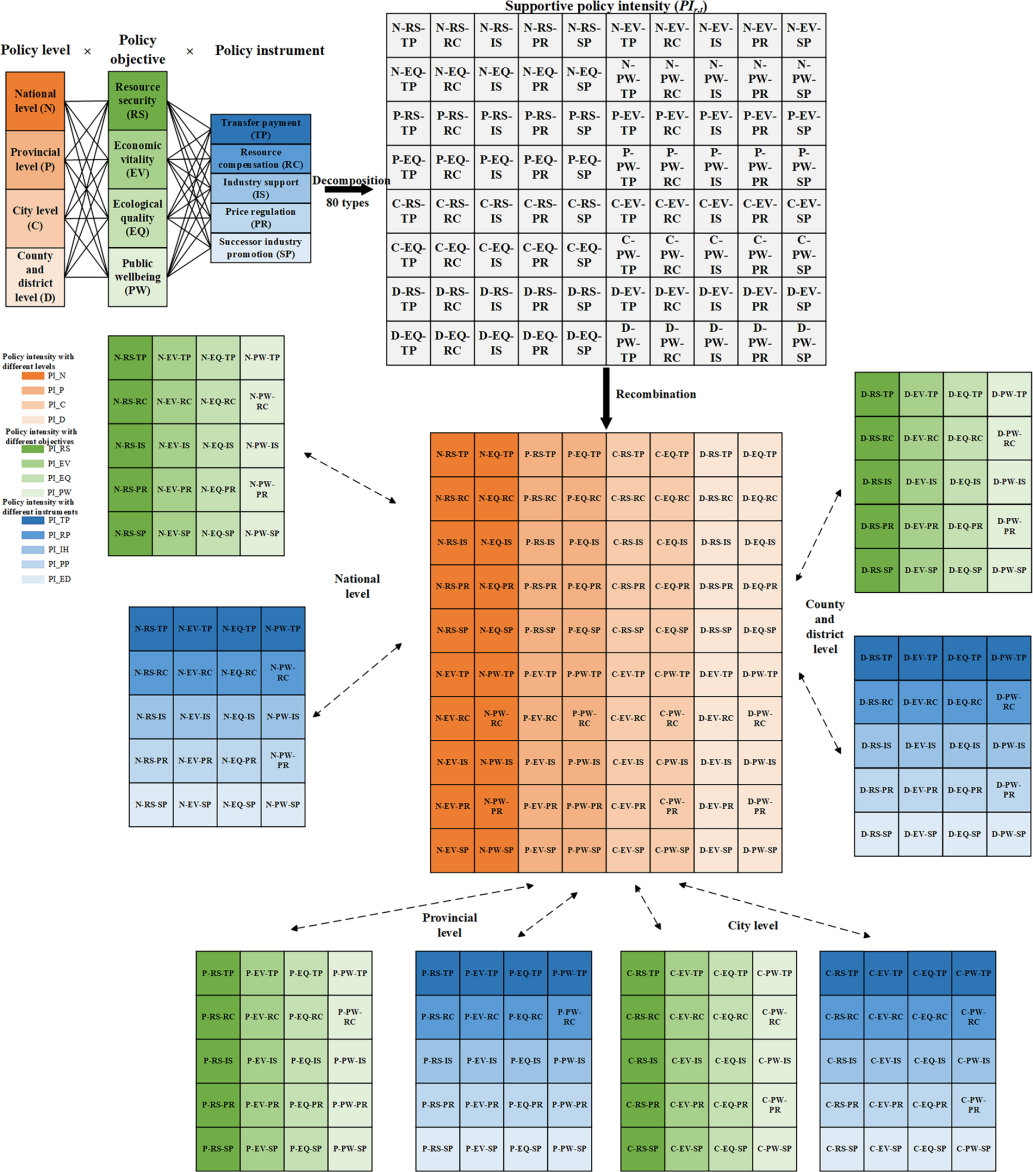
Aggregation structure of the supportive policy intensity index.

As shown in Fig. 2, the calculation framework encompasses three dimensions:

Policy level (*l*): national, provincial, prefectural (i.e., city)-level, and county/district levels;

Policy objective (*o*): resource security, economic vitality, ecological quality, and public wellbeing;

Policy instrument (*i*): transfer payments, resource compensation, industry support, price regulation, and successor industry promotion.

Each policy item is classified according to the occurrence of keywords corresponding to specific level, objective, and instrument within government work reports. The resulting dataset encompasses 80 combinations (4 levels × 4 objectives × 5 instruments), enabling flexible aggregation across different dimensions.

To obtain overall or dimension-specific policy intensity, Eq. (1) can be extended to the following summation forms:

2 $$P{I}_{r,t}=PI\_R{S}_{r,t}+PI\_E{V}_{r,t}+PI\_E{Q}_{r,t}+PI\_P{W}_{r,t}$$

3 $$P{I}_{r,t}=PI\_T{P}_{r,t}+PI\_R{C}_{r,t}+PI\_I{S}_{r,t}+PI\_P{R}_{r,t}+PI\_S{P}_{r,t}$$

where *PI*_*r,t*_ denotes the sum of policy intensity promulgated by region *r* in year *t*. From the perspective of policy level, *PI*_*r,t*_ is the sum of *PI_N*_*t*_, *PI_P*_*ρ,t*_, *PI_C*_*c,t*_, and *PI_D*_*d,t*_. *PI_P*_*ρ,t*_ represents the provincial-level intensity in province *ρ* in year *t*. *PI_C*_*c,t*_ represents the prefectural-level (city *c*) policy intensity in year *t*. *PI_D*_*d,t*_ refers to county- and district-level intensity in region *d* and year *t*. In Eq. (2), *PI*_*r,t*_ is summed by four different policy objectives. Specifically, *PI_RS*_*r,t*_, *PI_EV*_*r,t*_, *PI_EQ*_*r,t*_, and *PI_PW*_*r,t*_ are policy intensities respectively aiming at resource security, economic vitality, ecological quality, and public wellbeing promulgated by region *r* in year *t*. Finally, *PI*_*r,t*_ can also be disaggregated by policy instrument type using Eq. (3). Here, *PI_TP*_*r,t*_, *PI_RC*_*r,t*_, *PI_IS*_*r,t*_, *PI_PR*_*r,t*_ and *PI_SP*_*r,t*_ represent policy intensity of transfer payments, resource compensation, industry support, price regulation and successor industry promotion instruments, respectively, in region *r* during year *t*.

This hierarchical structure allows researchers to aggregate indices across geographical or thematic dimensions, thereby facilitating in-depth empirical analyses on the impact of supportive policy interventions in China’s resource-based regions.

### Manual labeling

To accurately measure policy intensity across policy levels, objectives, and instruments, all 3788 policy texts require manual annotation. The annotation process evaluates each text along three dimensions: policy level *L*_*r,t,l*_, policy objective *O*_*r,t,o*_, and policy instrument *I*_*r,t,i*_. Each dimension is rated on a four-point scale: Label 1 (weak intensity), Label 2 (medium intensity), Label 3 (high intensity), and Label 4 (highest intensity).

The labeling is based on the frequency of predefined keywords relevant to each dimension within the text. The specific scoring rules are as follows:

Label 1: keyword frequency = 0;

Label 2: keyword frequency = 1 or 2;

Label 3: keyword frequency = 3 or 4;

Label 4: keyword frequency > 4.

These manually annotated intensity scores form the basis for calculating the composite supportive policy intensity using Eq. (1).

### Supportive policy intensity integration

**Fig. 3:**
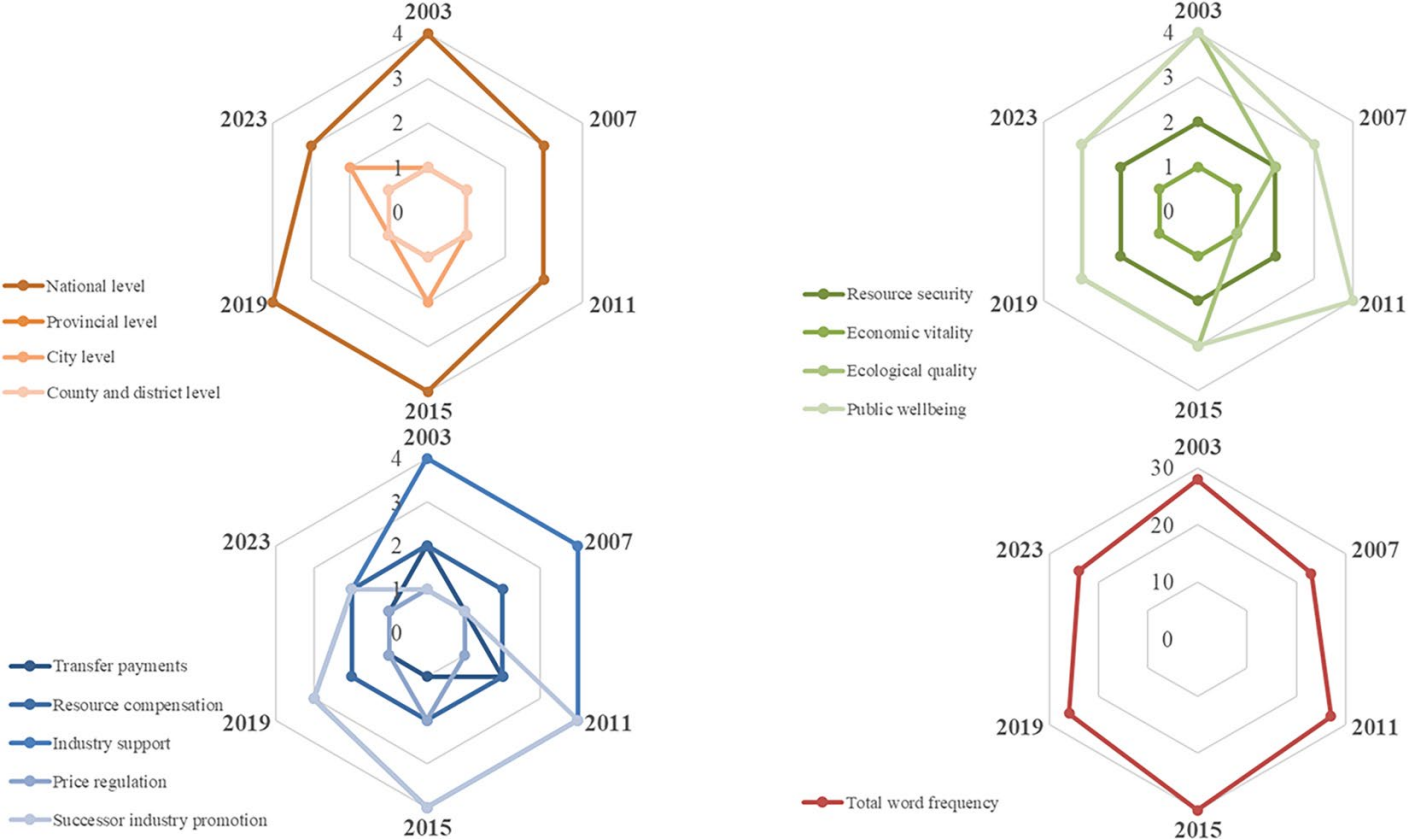
Radar chart of national-level supportive policy intensity across key dimensions.

Based on the manually annotated dataset, overall supportive policy intensity is calculated using Eq. (1) by integrating scores across three dimensions of policy level, objective, and instrument. As shown in Fig. 3, national-level policy intensity exhibits notable cyclical fluctuations during the study period, with distinct peaks in 2003, 2015, and 2019. These peaks correspond to the launch of the Northeast China Revitalization Strategy and the strengthening of green transition policies under the “dual carbon” target. Among policy objectives, ecological quality and public wellbeing have gained prominence since 2015, reflecting a shift from purely economic restructuring toward comprehensive environmental and social development goals. In terms of policy instruments, successor industry promotion and price regulation have become increasingly central, indicating a gradual transition from administrative directives to market-oriented and incentive-based policy tools. These dynamics demonstrate the evolving coordination between administrative levels and policy instruments in steering the green transformation of RBCs.

**Fig. 4:**
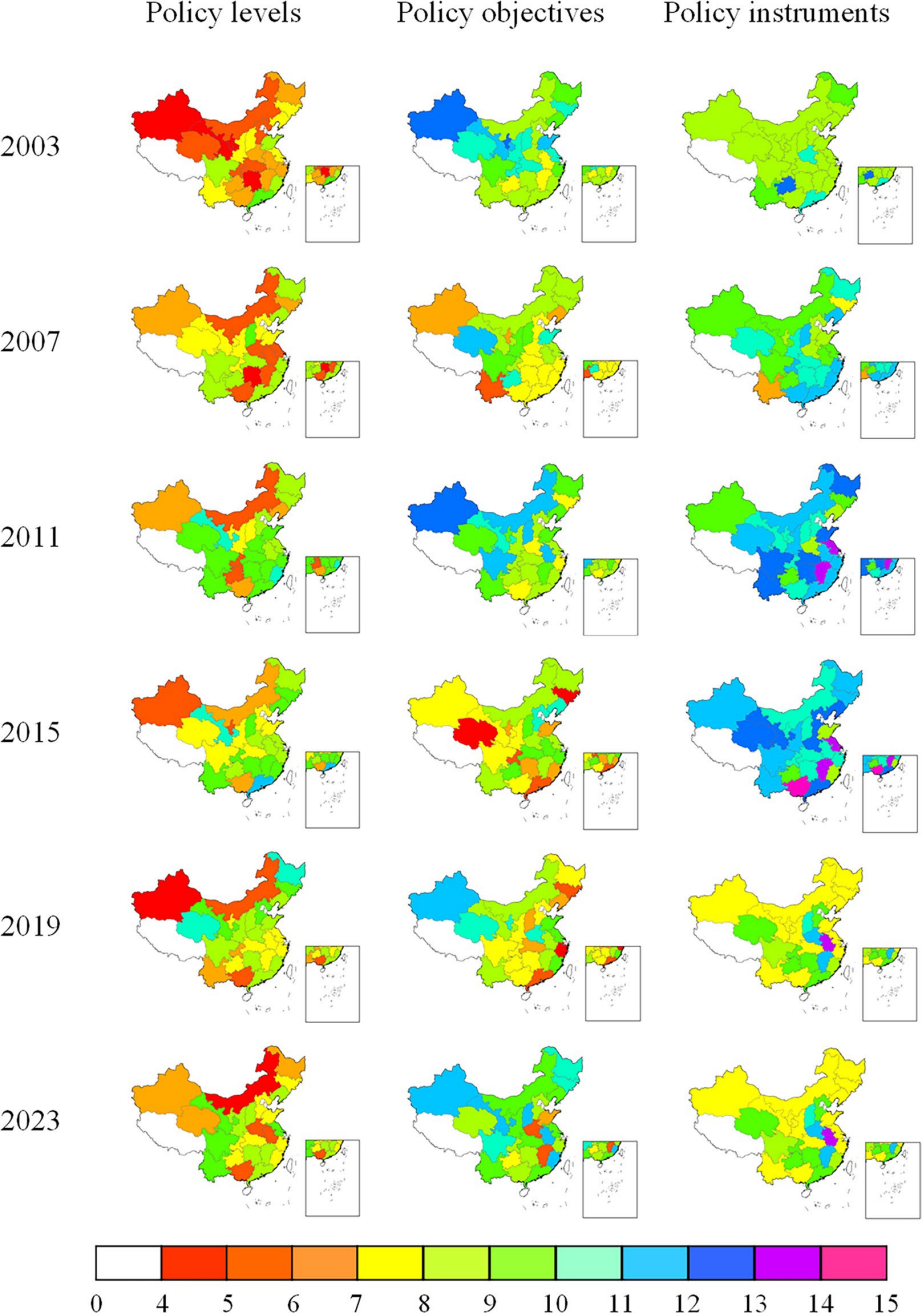
Heterogeneous supportive policy intensity across policy levels, temporal, and spatial dimensions at the provincial level.

**Fig. 5:**
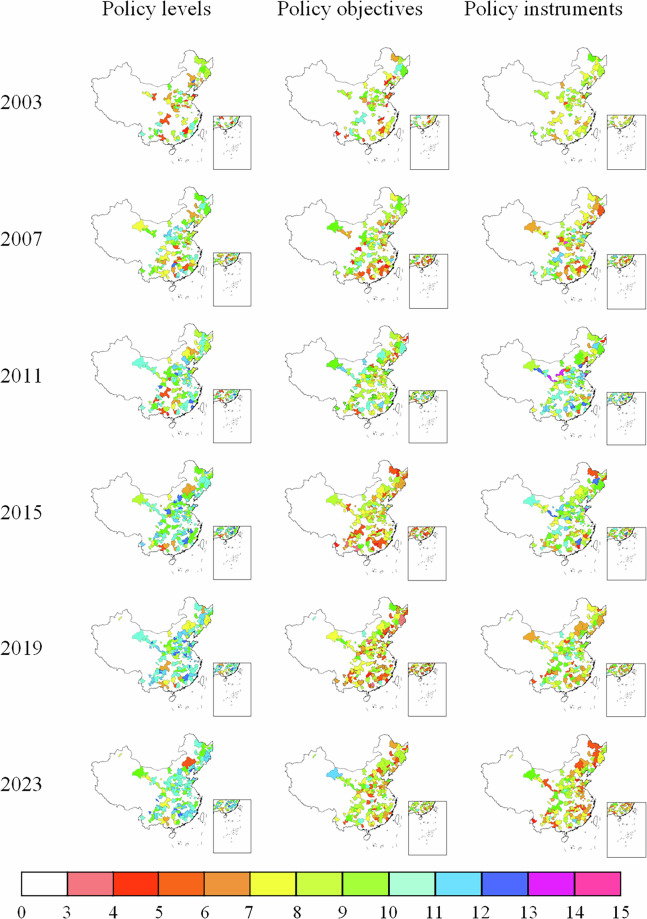
Heterogeneous supportive policy intensity across policy levels, temporal, and spatial dimensions at the prefecture level.

Figures 4, 5 show policy intensity heterogeneity across administrative levels, temporal, and spatial dimensions at the provincial and prefectural levels, respectively. Overall, prefecture-level policy intensity is generally lower than that of the provincial level, reflecting disparities in administrative capacity and policy-making authority. Nevertheless, several prefectural cities exhibit intensity levels comparable to their provincial counterparts, indicating strong local initiative in policy implementation.

It is worth noting that at the provincial level, Sichuan, Shanxi, and Guangdong provinces exhibit the highest average supportive policy intensity during the study period, suggesting more proactive and sustained commitment to regional green transformation.

Meanwhile, among the 168 resource-based prefectural cities, 19 cities attained the highest intensity level, distributed across multiple regions: Northern China (7 cities), Northwest China (4 cities), Eastern China (3 cities), Southwest, Central, and Western China (5 cities). This dispersed spatial pattern indicates that high-intensity policy support depends not merely on geographical location, but also on local development priorities, resource endowment structures, and alignment with upper-level policy agendas.

**Fig. 6:**
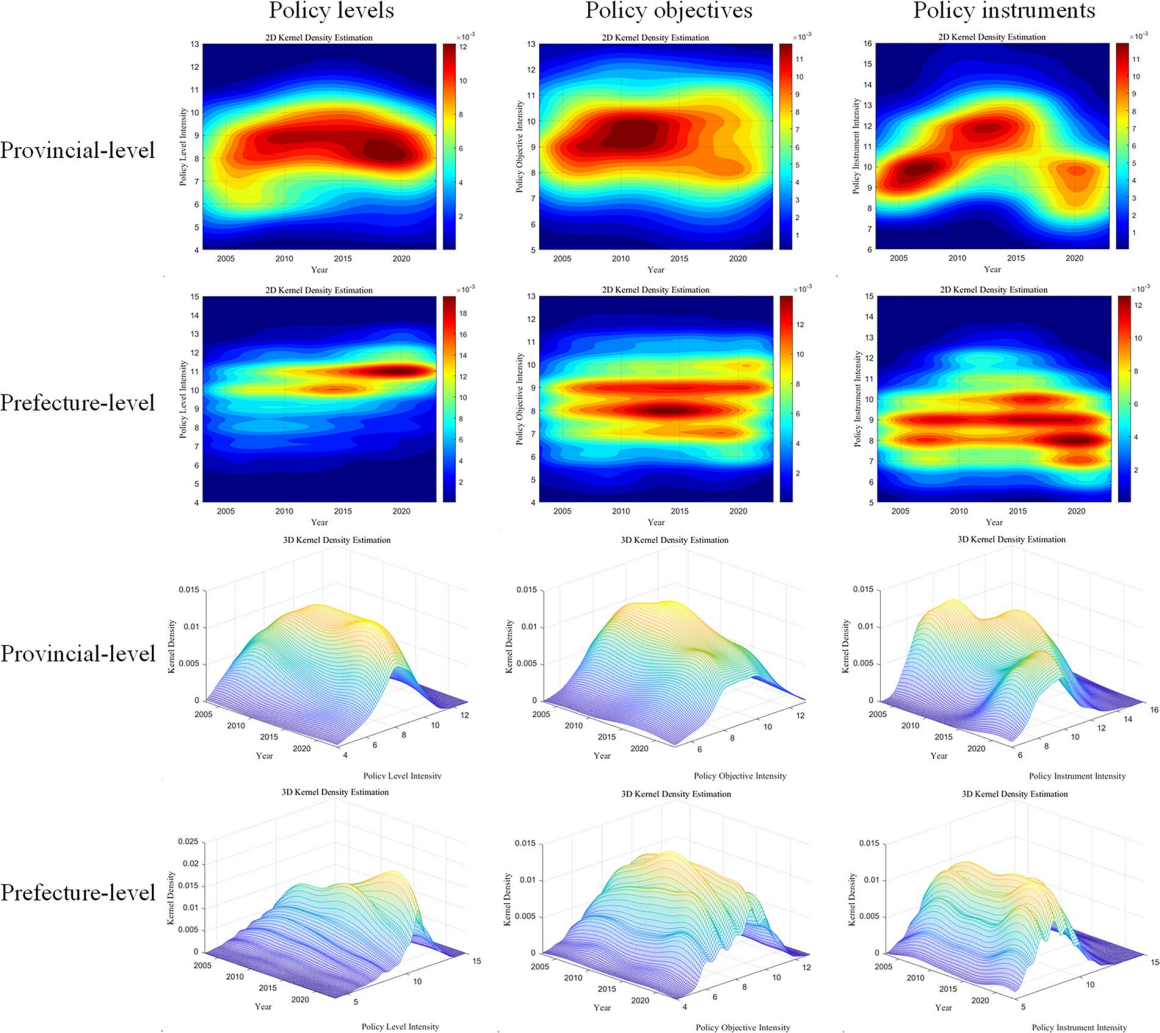
Kernel density estimation of supportive policy intensity at the provincial and prefectural levels.

Figure 6 presents the kernel density plots of supportive policy intensity at the provincial and prefectural levels, further disaggregated by policy levels, objectives, and instruments. Compared to simple descriptive statistics, kernel density estimation captures continuous distribution characteristics and identifies subtle temporal variations.

It can be seen from Fig. 6 that at the provincial level, the distribution curves are predominantly concentrated within higher policy intensity ranges, indicating that provincial governments generally adopt forceful and comprehensive measures to guide the green transformation of RBCs. At the prefectural level, policy levels remain concentrate in high-intensity ranges, while policy objectives cluster around medium intensity and policy instruments predominantly fall within the lower intensity ranges. This pattern may reflect operational constraints faced by prefectural governments: despite demonstrating strong commitment in policy articulation, implementation remains more conservative in terms of specific objectives and instruments.

Overall, provincial-level policies exhibit a uniformly high-intensity profile, whereas prefectural-level policies display a distinct pattern of high policy administrative level intensity, medium policy objective intensity, and low policy instrument intensity. This contrast highlights how multi-level governance may lead to attenuated policy intensity from provincial to prefectural levels, influenced by differential administrative authority, fiscal capacity, information access, and cross-regional coordination responsibilities.

**Fig. 7:**
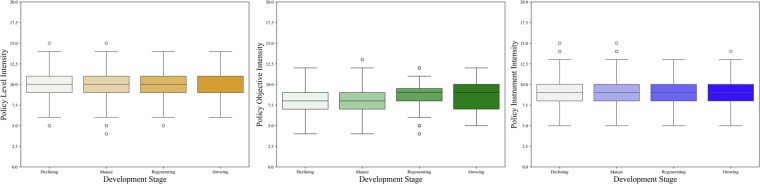
Box plots of policy level, objective, and instrument by development stage of RBCs.

In addition, to further explore differences in supportive policy intensity across city types, we categorize RBCs into four types based on their development stage: declining, mature, regenerating, and growing, following the *National Resource-Based City Sustainable Development Plan* (2013–2020). As presented in Fig. 7, the box plots indicate distinct patterns of four types of cities across three policy dimensions.

As for policy level, the median and interquartile ranges are relatively consistent across all four city types, though mature cities exhibit notably lower outliers, suggesting generally stable yet weaker policy engagement. In terms of policy objective, regenerating and growing cities show higher median values, with the latter demonstrating particularly wide dispersion, indicating substantial intra-group variability in policy prioritization. Regarding policy instrument, all groups maintain similar median and interquartile ranges, but declining and mature cities display higher outliers, implying occasional adoption of policy tools beyond the typical range. As such, these findings suggest that while overall policy intensity distributions are broadly comparable, specific development stages exhibit distinct variation patterns, potentially reflecting differences in administrative priorities, resource endowments, or transition pressures.

## Data Records

The dataset comprises one publicly available data file in the Figshare^44^ repository. To accommodate researchers from diverse disciplines and with varying analytical preferences, the dataset is provided in both Stata (.dta) and Excel (.xlsx) formats. The overall folder structure and content descriptions of the dataset are summarized in Table 3.

**Table 3:** Detailed information for data files.

Folders	Contents for datasets
Supportive policy intensity	Prefecture-level supportive policy intensity (.dta,.xlsx)
	Provincial-level supportive policy intensity (.dta,.xlsx)
	National-level supportive policy intensity (.dta,.xlsx)

### Supportive policy intensity

This folder contains Excel and Stata files at national, provincial, and prefectural levels. Each file includes three core variables: year, region (city or province name), and the intensity of 80 supportive policy mix indicators. All intensity values are numeric, with English variable names. These files are available in the folder of “Supportive policy intensity”.

## Technical Validation

### Collection of datasets

The dataset of supportive policy documents from 2003 to 2023 was compiled by systematically collecting government work reports from the official websites of the central, provincial, and prefecture-level governments in China. To ensure reliability and accuracy, the data collection process involved multiple stages of manual screening, cross-checking, and annotation. Each policy text was carefully reviewed and rigorously screened to extract information pertaining to RBCs supportive policies. These procedures improve the overall validity and precision of the dataset, ensuring that it accurately reflects policy content across different administrative levels.

### Comparison with existing studies

Due to the lack of publicly available datasets specifically focused on supportive policy intensity for RBCs, direct comparisons with existing datasets are currently not feasible. However, relevant methodological precedents can be drawn from other policy domains. For instance, Zeng *et al*. developed a supportive policy intensity index for small and medium-sized enterprises (SMEs) in Zhejiang Province from 2018 to 2022, based on an analysis of local government policy documents^45^. Their approach, which involves keywords identification and policy content categorization to quantify policy support, shares methodological similarities with our study.

Although both studies rely on policy text extraction and classification, our dataset advances this approach in several key aspects. First, it encompasses four administrative levels, enabling multi-tiered policy analysis. Second, it integrates four dimensions of policy objectives and five types of policy instruments, offering a more nuanced expressions for policy design. Third, it covers a 21-year timeframe, providing a longitudinal perspective that has been largely absent in previous research. Finally, by focusing on China’s RBCs, a type of city that faces unique developmental challenges and possesses strategic significance, this dataset addresses a significant gap in the study of region-specific supportive policy systems.

Table 4 summarizes the dataset used in this study alongside several representative datasets from related fields. The comparison highlights the study’s broader coverage, finer granularity, and wider time frame, underscoring its contribution to strengthening the empirical foundation of policy intensity research.

**Table 4:** Overview of existing datasets related to policy intensity measurement.

Dataset name	Level	Granularity	Content	Year coverage	Source
Multi-level supportive policy dataset for China’s resource-based cities	Multi-level	Annual	Supportive policy intensity of RBCs based on government work reports	2003–2023	Our data
China’s low-carbon policy intensity dataset from national- to prefecture-level over 2007–2022	Multi-level	Annual	Low-carbon policy intensity index for China’s manufacturing industries covering national-, provincial- and prefecture-level policies	2007–2022	Dong *et al*.^34^
Policy involvement and policy consistency identification of supportive policies for SMEs	Company	Annual	Quantitative evaluation of supportive policies for SMEs in Zhejiang	2018–2022	Zeng *et al*.^45^
Balancing economic growth and environmental conservation: assessing supportive policies in resources-based cities in China	National	Annual	Evaluation of supportive policies based on dummy variables	2013–2020	Liu *et al*.^19^
Toward a comparative measure of climate policy output	National	Annual	Index of climate policy activity based on countries’ policy portfolios	1998–2010	Schaffrin *et al*.^31^
Metricizing policy texts: Comprehensive dataset on China’s agri-policy intensity spanning 1982–2023	National	Annual	Evaluation of agricultural policy intensity at the national level in China	1982–2023	Wu *et al*.^35^
China’s environmental policy intensity for 1978–2019	National	Annual	Evaluation of environmental policy based on government work reports	1978–2019	Zhang *et al*.^30^

## Usage Notes

### Interpretation of policy intensity

In practical applications, the interpretation of policy intensity should shift from the perspective of policymakers to that of policy recipients. The total RBCs supportive policy intensity received by region *r* in year *t*, denoted as *PI_all*_*r,t*_, aggregates all relevant policies across administrative levels. Similarly, *PI_all*_*ρ,t*_ represents the total policy intensity received by province *ρ* in year *t*; *PI_all*_*c,t*_ represents the policy intensity received by resource-based city *c* in year *t*; *PI_all*_*d,t*_ represents the policy intensity received by county/district *d* in year *t*.

Given China’s multi-tiered governance structure, the central government delegates policy targets to provinces, which subsequently allocate these targets to lower administrative levels through target responsibility system (mubiao zerenzhi) and performance-based management. Therefore, local governments act as both implementers and recipients of multi-tiered policy mandates.

To illustrate, when focusing on outcomes at the county/district-level, the appropriate policy intensity metric is *PI_all*_*d,t*_, defined as the sum of county/district-level policies (*PI_D*_*d,t*_).

Policies from higher administrative levels are also included, namely: national (*PI_N*_*t*_), provincial (*PI_P*_*ρ,t*_), and prefecture-level (*PI_C*_*c,t*_), as aggregated below:

4 $$\begin{array}{rcl}PI\_al{l}_{d,t} & = & PI\_{D}_{d,t}+PI\_aboveDistric{t}_{d,t}\\  & = & PI\_{D}_{d,t}+PI\_{C}_{c,t}+PI\_{P}_{\rho ,t}+PI\_{N}_{t}\end{array}$$

Similarly, for prefecture-level analysis:

5 $$PI\_al{l}_{c,t}=PI\_{C}_{c,t}+PI\_{P}_{\rho ,t}+PI\_{N}_{t}$$

When examining policy impacts, researchers may choose to include both *PI_D*_*d,t*_ and *PI_aboveDistrict*_*d,t*_ to distinguish between local initiatives and those imposed by higher administrative levels.

### Limitations and future work

This study has several limitations. First, the dataset is constrained by the public availability of government work reports. Not all policy documents have been fully disclosed, and the intensity if non-public or partially released policies may be underestimated.

Second, this study focuses primarily on the policy issuance phase (i.e., policy release), while policy implementation and enforcement, critical factors shaping policy effectiveness, are not directly assessed. Future research could extend this work by incorporating policy execution data, such as budgetary expenditures, implementation reports, or third-party evaluations, to investigate gaps between policy design and practice.

Furthermore, it is important to explore the varying effectiveness of multi-level policies across different domains (e.g., ecology, economy, society), though such an analysis falls beyond the scope of this study. Future research should continue to advance this line of inquiry to develop a deeper understanding of the mechanisms underlying the transformation of RBCs.

## Data Availability

The constructed supportive policy intensity index of RBCs has been uploaded to Figshare^44^. Data were processed with Stata 17 and Python 3.12.3. The code used to calculate supportive policy intensity of RBCs is also available in the Figshare repository. We share the dataset and replication codes to enhance data transparency and computational reproducibility, thereby facilitating further research and exploration by users.
